# Genetic Passive Immunization with Adenoviral Vector Expressing Chimeric Nanobody-Fc Molecules as Therapy for Genital Infection Caused by *Mycoplasma hominis*

**DOI:** 10.1371/journal.pone.0150958

**Published:** 2016-03-10

**Authors:** Daria A. Burmistrova, Sergey V. Tillib, Dmitry V. Shcheblyakov, Inna V. Dolzhikova, Dmitry N. Shcherbinin, Olga V. Zubkova, Tatiana I. Ivanova, Amir I. Tukhvatulin, Maxim M. Shmarov, Denis Y. Logunov, Boris S. Naroditsky, Aleksandr L. Gintsburg

**Affiliations:** 1 Department of Immunobiotechnology, Gamaleya Research Center of Epidemiology and Microbiology, Moscow, Russia; 2 Department of Molecular Biotechnology, Institute of Gene Biology, Moscow, Russia; 3 Department of Cellular Microbiology, Gamaleya Research Center of Epidemiology and Microbiology, Moscow, Russia; 4 Department of Molecular Biotechnology, Gamaleya Research Center of Epidemiology and Microbiology, Moscow, Russia; 5 Director of Gamaleya Research Center of Epidemiology and Microbiology, Moscow, Russia; Instituto Butantan, BRAZIL

## Abstract

Developing pathogen-specific recombinant antibody fragments (especially nanobodies) is a very promising strategy for the treatment of infectious disease. Nanobodies have great potential for gene therapy application due to their single-gene nature. Historically, *Mycoplasma hominis* has not been considered pathogenic bacteria due to the lack of acute infection and partially due to multiple studies demonstrating high frequency of isolation of *M*. *hominis* samples from asymptomatic patients. However, recent studies on the role of latent *M*. *hominis* infection in oncologic transformation, especially prostate cancer, and reports that *M*. *hominis* infects *Trichomonas* and confers antibiotic resistance to Trichomonas, have generated new interest in this field. In the present study we have generated specific nanobody against *M*. *hominis* (aMh), for which the identified target is the ABC-transporter substrate-binding protein. aMh exhibits specific antibacterial action against *M*. *hominis*. In an attempt to improve the therapeutic properties, we have developed the adenoviral vector-based gene therapy approach for passive immunization with nanobodies against *M*. *hominis*. For better penetration into the mucous layer of the genital tract, we fused aMh with the Fc-fragment of IgG. Application of this comprehensive approach with a single systemic administration of recombinant adenovirus expressing aMh-Fc demonstrated both prophylactic and therapeutic effects in a mouse model of genital *M*. *hominis* infection.

## Introduction

Mycoplasmas are atypical bacteria that lack a cell wall, a feature that complicates both: diagnosis and treatment of mycoplasma infection. Mycoplasmas are highly prevalent in cervicovaginal cultures of sexually active women as opportunistic bacteria associated with undesirable gynecologic and reproductive events. *Mycoplasma hominis* is an etiologic agent of non-gonococcal urethritis, cervicitis, and endometritis, and infertility in both immunosuppressed and immunocompetent individuals [1,2].

*M*. *hominis* usually colonizes the genitourinary tract in a nonvirulent manner, but may cause postoperative, postpartum and posttraumatic infections in various organ systems: blood, wounds, central nervous system, joints and respiratory tract [3–8]. Moreover, infection occurring during pregnancy may lead to chorioamnionitis, subsequent pregnancy complications and neonatal infection [9]. In addition, previous observations link inflammation caused by *M*. *hominis* infection to cell transformation, genomic instability, resistance to apoptosis, and cancer development [10–12].

*M*. *hominis* surface lipoproteins play an important role in colonization and adaptation to ecologic niches associated with mucosal tissues, especially those of the genitalia. Studies of *M*. *hominis* pathogenicity have identified some surface proteins that could be targeted therapeutically, including members of the ABC transporter system [13]. ABC transporters represent one of the largest superfamily of membrane transport complexes which take up a variety of nutrients and extrude drugs and metabolic waste. More than half of the membrane proteins of mycoplasmas belong to the ABC transporter family [14,15]. Different ABC transporters have important roles as vital and virulence factors of mycoplasmas and could be novel therapeutic targets.

Treatment of mycoplasma infection comprises antibiotics alone or in combination with immunomodulatory drugs. At the same time development of antibiotic resistance among mycoplasmas restricts the efficacy of antibiotics [16,17].

Antibodies, unlike antibiotics, can bind a specific pathogen and may have various modes of antibacterial activity: neutralization, complement-mediated bactericidal activity, opsonophagocytosis, direct bactericidal activity and anti-virulence activity [18] [19]. Canonical antibodies, however, have some limitations. Therefore, the development of various other strategies to generate antibody fragments (*i*.*e*. Fab, scFv, VH, and VL) has become more widespread.

Nanobodies represent the smallest known intact antigen-binding fragments derived from heavy-chain antibodies devoid of light chains, which occur naturally in Camelidae and sharks [20]. Nanobodies combine the advantages of antibodies with important features of single-gene coding proteins. Like conventional antibodies, nanobodies exhibit high target specificity, high target affinity and low inherent toxicity. However, nanobodies can easily access to receptor clefts through unusual conformation of CDR loops. Nanobodies are currently a topic of high research interest for various applications due to their ability to target unique epitopes that are less well targeted by conventional antibodies. Therefore, nanobodies form the basis of a new generation of therapeutic antibodies cause of their unique structural and functional properties [21–27].

Practical use of nanobody fragments is limited by problems related to rapid blood clearance and poor retention time at the target which result in the need for frequent delivery of these molecules [28]. *In vivo* production with gene therapy vectors results in effective and persistent levels of nanobodies [29,30]. The unique ability of human adenovirus serotype 5 (Ad5) to accomplish efficient transduction has allowed for the use of Ad5-based vectors for a range of gene therapy applications [31]. Furthermore, nanobodies may be encoded by a single polypeptide gene and thus may be used as building blocks for genetic engineering manipulation including construction of fusion proteins with different functional domains such as an Fc domain. Fc-engineering can promote both stable immune-mediated reactions and receptor-mediated specific distribution in tissues [32].

Here we report the use of an alternative strategy for the treatment of genital infection caused by *M*. *hominis* through genetic passive immunization with nanobody-Fc chimeric antibodies that comprise a mouse FcG2a domain fused with a specific nanobody produced by recombinant adenovirus-based vector.

The aim of this study was to develop a novel therapeutic approach for *M*. *hominis* infection using adenoviral vector-based passive immunization with a nanobody-Fc chimeric drug. This comprehensive approach combines the advantages of gene-therapy, specific nanobody properties and Fc-mediated reactions.

## Materials and Methods

### Ethics Statement

All animal work was undertaken in strict accordance with the recommendations in the National Standard of the Russian Federation GOST R 53434–2009. The procedures used were approved by the Gamaleya Research Center of Epidemiology and Microbiology Institutional Animal Care and Use Committee (IACUC) and were performed under Protocols #Imb-2013-011; #Imb-2014-048 and #Imb-2014-049. A five-year-old male two-hump camel (*Camelus bactrianus)* was housed at Scientific-Experimental Base "Chernogolovka" of Institute of Problems of Ecology and Evolution named after A. N. Severtsov of Russian Academy of Sciences (Chernogolovka, Russia) and used for immunization and blood collection (under Protocol #Imb-2013-011). Six-week-old female DBA/2 mice (weighing 18–20 g) were purchased from “Pushchino breeding facility” (Pushchino, Russia) accredited by Association for Assessment and Accreditation of Laboratory Animal Care (AAALAC International) and maintained at the central animal facility at the Gamaleya Research Center of Epidemiology and Microbiology. The experiments with DBA/2 mice were approved by the Gamaleya Research Center of Epidemiology and Microbiology Institutional Animal Care and Use Committee (IACUC) and were performed under Protocols #Imb-2014-048 and #Imb-2014-049.

### Mycoplasma strains and cultivation conditions

Reference strain H-34 of *M*. *hominis*, *M*. *arginini*, *M*. *fermentans*, *M*. *pneumoniae*, *M*. *orale*, *M*. *arthritidis*, *Ureaplasma urealiticum*, and *M*. *hominis* clinical isolates 299, 312, X-37, 153 were provided by I.V. Rakovskaya (Gamaleya Institute of Epidemiology and Microbiology, Russian Ministry of Health, Moscow, Russia); mouse-adapted clinical isolate *M*. *hominis* 1862.3 was provided by V.N. Lazarev (Institute of Physicochemical Medicine, Russian Ministry of Health, Moscow, Russia).

*M*. *hominis* broth culture was performed at 37°C in IST-2 medium (BioMérieux, Marcy l’Etoile, France) containing a colorimetric indicator of mycoplasma growth. Seeding was performed using standard microbiologic techniques with cultivation under anaerobic conditions at 37°C with an abundance of CO_2_.

### Color-change unit assay to determine the mycoplasma titer

The mycoplasma titer was determined using a modified color-change unit (CCU) assay: 10-fold dilutions of samples were inoculated in 50 μl IST2 medium (BioMérieux, Marcy l’Etoile, France), samples were covered with mineral oil and incubated in microtiter strips (BioMérieux, Marcy l’Etoile, France) for 48 h; the last dilution that caused a change in the color of the medium corresponded to the titer of the start sample. We used both a CCU assay and real-time polymerase chain reaction (PCR) to more accurately determine the titer in mouse vaginal washes.

### Real-time PCR to determine the mycoplasma titer

For more accurate determination of the *M*. *hominis* titer, we used the AmpliSens^®^ Mycoplasma hominis-screen-titer kit (InterLabService Ltd, Moscow, Russia), including both DNA-isolation reagents and amplification solutions. Amplified *M*. *hominis* DNA was detected with FAM fluorescence and a calibration curve was created using samples obtained from serial dilutions of *M*. *hominis* broth culture and processed in parallel. Amplification was performed according to the supplier’s protocol for the CFX96 Touch Real-Time PCR Detection System (Bio-Rad, Singapore).

### Preparation of lipid-associated membrane proteins

Lipid-associated membrane proteins (LAMPs) were isolated from 100 ml *M*. *hominis* H-34 broth culture by fractionation in Triton X-114 (A3848, PanReacApplyChem). *M*. *hominis* pellets were suspended in 10 ml buffer containing 2% Triton X-114, 10 mM Tris-HCl pH = 7.4, and 150 mM NaCl. After 2 h incubation on ice with rotation for mycoplasma lysis, the cell debris was pelleted at 10,000g for 15 min. The supernatant was warmed to 37°C and incubated for phase separation (~20 min). For better micelle separation, the supernatant was centrifuged at 1000g for 5 min, the aqueous layer was removed and replaced with an equal volume of 10 mM Tris-HCl, 150 mM NaCl pH = 7.4 and the supernatant was placed in an ice bath for 20 min. Phase separation was repeated three times. LAMPs were precipitated with ethanol and dissolved in phosphate buffered saline (PBS), protein concentration were measured by Quick Start^™^ Bradford assay (Bio-Rad, Hercules, CA, USA).

### Camel immunization with LAMPs from *M*. *hominis*

A two-hump camel (*C*. *bactrianus*) was immunized (5 subcutaneous immunizations) with *M*. *hominis* H-34 LAMPs (1 mg/injection) in Freund’s adjuvant (complete for the first injection and incomplete for the remaining four injections). The second injection was administered 3 weeks after the first, and the remaining three injections were given at 14-days intervals. Blood samples (150 ml) were obtained 5 days after the last injection. An equal volume of phosphate-buffered saline (PBS) containing heparin (100 U/ml) and EDTA (3 mM) was added to the blood to prevent clotting. The immunization and blood collection protocol was approved by the Gamaleya Research Center of Epidemiology and Microbiology Institutional Animal Care and Use Committee (IACUC) and were performed under Protocol #Imb-2013-011.

### Selection of nanobodies by phage display

A library of nanobody-coding sequences was constructed, and nanobodies against *M*. *hominis* LAMPs were selected by M13-phage display technology according to the previously described procedure [33]. The entire repertoire of the variable domains of *Camelidae*-specific heavy chain-only antibodies from peripheral blood B-lymphocytes of the immunized camel were cloned into pHEN4 phagmid vector. Recombinant virions were obtained by packaging with M13KO7 helper phage. One hundred nanograms of LAMPs in coating buffer (0.1 M NaHCO3 pH 9.6) were coated in microtiter plates at 4°C overnight. After blocking the plate with 1% BSA in PBS, total 1011 phages in PBS with 3% dry milk were added into each well and incubated for 2 h at room temperature. Unbound phages were removed by washing with 0.1% Tween20 in PBS six times and bound phages were eluted by pH treatment with a freshly prepared solution of 1.4% triethylamine. Panning was repeated three times.

The production of nanobodies in bacterial periplasmic space and indirect enzyme-linked immunosorbent assay (iELISA)-based analysis of the ability of the nanobodies to recognize the given antigen were performed using the previously described techniques [33,34].

### Expression and purification of nanobodies

The cDNA sequences of the selected nanobodies were subcloned (by conventional or PCR cloning) into the pHEN6 expression plasmid [35] together with the pelB leader sequence (for periplasmic production), camel upper hinge (the longest hinge variant), and two short tag sequences (hemagglutinin [HA]-tag and (His)6-tag) at the C-terminus coding region as described previously [36]. The plasmids were transformed into *Escherichia coli* BL21 cells (New England BioLabs, Ipswich, MA, USA) for nanobodies expression and purification. Protein expression was induced by the addition of 1 mM isopropyl-d-1-thiogalactoside. After 5 to 7 h of induction at 37°C, the cells were harvested by centrifugation, and the nanobodies were purified from the periplasmic extract using Ni—NTA agarose and the QIAExpressionist purification system (Qiagen Corporation, Boston, MA, USA). The eluted fraction was concentrated to a final concentration of approximately 1 to 5 mg/ml in Amicon 10 kDa ultrafiltration devices (Merck Millipore Ltd., Carrintwohill, Co. Cork, Ireland), and 0.5 to 2 ml was loaded into a Sephacryl S-100 HR gel filtration column. Size exclusion chromatography was performed using the BioLogic LP System (Bio-Rad, Hercules, CA, USA) at a constant flow rate of 0.3 ml/min with PBS as a running buffer. The nanobodies had an expected molecular weight of approximately 20 kDa, and were eluted as a single peak with an elution volume of 65 to 75 ml (between ovalbumin [45 kDa] and lysozyme [14.4 kDa]).

### Experimental *M*. *hominis* infection of A549 cell line

A549 adenocarcinomic human alveolar basal epithelial cell line was purchased from Russian collection of vertebrate cell lines (Saint Petersburg, Russia). A549 cells were passaged two times in presence of *M*. *hominis* H-34 10^4^ CFU/ml in cell culture medium DMEM with 10% fetal bovine serum, and then after additional three passages in mycoplasma-free medium, the cells were tested using a MycoAlert^™^ Mycoplasma Detection Kit (Lonza, Rockland, ME, USA) and used for a cell-based ELISA assay.

### Cell-based ELISA

*M*. *hominis*-infected and control A549 cells were plated at a density of ~5 x 104 cells/ml in a 96-well plate and incubated for 18 h. Attached cells were fixed with 2% paraformaldehyde in PBS for 15 min at room temperature and washed two times with PBS. Plates were blocked with 2% (w/v) ovalbumin in PBS. HA-tagged nanobodies were then added (~1 μg/ml in PBS and 0.05% Tween-20 [PBS-T]) and the plates were incubated at 37°C for 1 h in a shaker incubator. The plates were washed three times with PBS-T and then incubated with anti-HA antibody-horseradish peroxidase conjugate, clone HA-7 (Sigma-Aldrich, St. Louis, MO, USA). Following the washing procedures described above, the antibody was detected with 3,3,5,5-tetramethylbenzidine substrate for 10 min and the reaction was stopped with 1 M H2SO4. Samples were analyzed in triplicate, and absorbance was measured at 450 nm.

### Antigen precipitation

For antigen precipitation, the aMh nanobodies were covalently coated onto the surface of magnetic NH2 beads (Sileks, Moscow, Russia). Then, 50 μg LAMPs were incubated with the beads suspension at 37°C for 1 h, and unbound proteins were washed 10 times with PBS. Magnetic beads with precipitated protein were boiled directly in SDS-PAGE sample buffer with DTT and loaded on gels. Proteins with mobility between 37 and 50 kDa was eluted from gel overnight by diffusion in PBS (~10 gel volume of PBS) and used for second round of precipitation. After staining the electrophoresis gel, the protein band was cut and used for identification using matrix-assisted laser desorption/ionization mass spectroscopy (MALDI).

### aMh-Fc fusion protein expression and purification

The nucleotide sequence encoding selected nanobody, named aMh, was subcloned with mouse Fc domain IgG2a in pSh-CMV-PLAP vector under the control of a CMV promoter in-frame with the PLAP signal peptide sequence (PLAP; DDBJ/EMBL/GenBank accession no. M13077). The plasmid vector was transformed into the A549 cell line using MetafectinPro (Biontex, Planegg, Germany) according to the supplier’s protocol. Transfected A549 cells were plated at a density of ~5 x 10^4^ cells/ml in serum-free DMEM. After 48 h, the recombinant aMh-Fc fusion protein was purified from culture medium using protein A—sepharose (Sigma-Aldrich, St. Louis, MO, USA) according to the supplier’s protocol for batch purification. The purified aMh-Fc was evaluated by 12% SDS-PAGE under reducing (sample buffer with DTT) and non-reducing (sample buffer without DTT) conditions to verify dimer formation, using mouse anti-Spt16 clone 8D2 (BioLegend, San Diego, CA, USA) as a control.

### SDS-PAGE and Western blotting

To verify dimer formation, purified aMh-Fc was separated on 12% Laemmli SDS-PAGE under non-reducing conditions (Laemmli loading buffer without reducing agent). For other experiments, sample buffer was supplemented with reducing agent, DTT (standard Laemmli loading buffer). Samples were loaded with concentrations 0.1–20 μg/well. After electrophoresis, protein bands were stained with EZBlue^™^ Coomassie Brilliant Blue G-250 colloidal protein stain (Sigma-Aldrich, St. Louis, MO, USA) or transferred to a nitrocellulose membrane using a Trans-Blot Turbo System (Bio-Rad, Hercules, CA, USA). The nitrocellulose membrane was blocked with 5% skim milk in PBS-T for 1 h at 4°C, washed, and incubated with antibody according to the supplier’s instructions. Working concentration of nanobodies in screening experiments was 1 mkg/ml, incubation condition– 16 hours at +4°C. As a secondary antibody for nanobodies detection, we used anti-HA antibody clone 3F10 horseradish peroxidase conjugate (Roche, IN, USA); as a secondary antibody for aMh-Fc detection, we used polyclonal goat anti-mouse IgG (Fc specific) antibody horseradish peroxidase conjugate (Sigma-Aldrich, St. Louis, MO, USA). Clarity ECL Western Blotting Substrate (Bio-Rad, Hercules, CA, USA) was used for detection.

### Surface plasmon resonance imaging

The binding of aMh-Fc to antigen was determined by surface plasmon resonance (SPR) using a Biacore 3000 (GE Healthcare, Uppsala, Sweden). Polyclonal rabbit anti-mouse IgG antibodies were immobilized at amount of 15000 RU in 10 mM acetate buffer pH = 5.0 on a CM5 sensor chip using the amine coupling kit and mouse antibody capture kit supplied by the manufacturer (GE Healthcare, Uppsala, Sweden). Kinetic was determined using various concentrations of antigen with regeneration solution 10mM glycine-HCl pH 1.7. Analyses were performed at 25°C in 10 mM HBS-EP running buffer (HEPES-buffer saline containing 150 mM NaCl, 3 mM EDTA, and 0.005% surfactant P20, pH 7.4) at a flow rate of 30 μl/min. Calculations were performed using BiaEvaluation software (GE Healthcare, Uppsala, Sweden) with reference-subtracted fitting.

### Evaluation of aMh-Fc inhibitory effect on *M*. *hominis*

*M*. *hominis* 1862.3 culture in IST2-medium were mixed with various concentrations of aMh-Fc or control mouse IgG2a antibody—anti-Spt16 clone 8D2 (BioLegend, San Diego, CA, USA). The titer of living mycoplasma cells was then determined using a CCU assay.

### Construction and production of recombinant adenoviruses

The nucleotide sequence encoding aMh was subcloned with the mouse Fc domain IgG2a in pSh-CMV-PLAP vector under control of a CMV promoter in-frame with the PLAP signal peptide sequence. To produce control rAd, the same nucleotide sequence was subcloned in the pSh-CMV-PLAP vector together with a sequence of the ILZ trimerizing domain and HA-tag. The AdEasy Adenoviral Vector System (Stratagene, La Jolla, CA, USA) was used to construct the recombinant adenovirus vectors (rAds) according to the manufacturer’s instructions. The rAd with the E1 region replaced with a transgene-free expression cassette (Ad-null) was used as a control. HEK293 human embryonic kidney cell line was purchased from Russian collection of vertebrate cell lines (Saint Petersburg, Russia). rAds were grown in HEK-293 cells and purified by double cesium chloride gradient centrifugation. The titers were determined by the plaque formation technique in HEK-293 cell culture. Particle size of virus suspension was determined using the Zetasizer Nano ZS (Malvern, Worcestershire, UK).

### Antibody titer determination in serum and vaginal washes

The DBA/2 mice were injected into the ophthalmic venous sinus with 10^7^ plaque-forming units of rAd5-CMV-PLAP-aMh-FcG2a or rAd5-CMV-PLAP-aMh-ILZ-HA previously dialyzed against PBS. As a control, we used mice injected with PBS. At 2 days (48 h), 7 and 14 days after the inoculation, serum samples and vaginal washes were collected, separated from cell debris by centrifugation, and serial sample dilutions were used for titration on ELISA with LAMPs or bovine serum albumin coated at a concentration of 100 ng/well. As a secondary antibody, we used polyclonal goat anti-mouse IgG (Fc specific) antibody horseradish peroxidase conjugate (Sigma-Aldrich, St. Louis, MO, USA) or anti-HA high affinity antibody (clone 3F10) (Roche, Indianapolis, IN, USA). The rAds injection and sample collection protocol was approved by the Gamaleya Research Center of Epidemiology and Microbiology Institutional Animal Care and Use Committee (IACUC) and was performed under Protocol # Imb-2014-048.

### Experimental genital infection and *in vivo* experiments

Beta-estradiol (Sigma-Aldrich, St. Louis, MO, USA) at a dose of 0.5 mg/kg was subcutaneously injected into DBA/2 mice to synchronize the estrous cycle, and the treatment was repeated at 1-week intervals. The mycoplasma suspension in PBS (5x10^6^ in 50 μl) was intravaginally administered using an Eppendorf automatic pipette immediately after the second injection of estrogen.

For *in vivo* experiments with rAds, mice were injected into the ophthalmic venous sinus with 10^7^ plaque-forming units of rAds previously dialyzed against PBS. In the “prophylactic” scheme, the rAds inoculation was performed 24 h before mycoplasma administration and in the “therapeutic” scheme the rAds inoculation was performed 5 days after mycoplasma administration.

Vaginal washes were collected at selected time-points with 50 μl PBS using an automatic pipette and divided into two parts: one for inoculation into IST2 medium and the other for DNA isolation and real-time PCR. The mycoplasma titer was determined using both a CCU-assay and real-time PCR.

The injection of estrogen, rAds administration and sample collection were approved by the Gamaleya Research Center of Epidemiology and Microbiology Institutional Animal Care and Use Committee (IACUC) and were performed under Protocol #Imb-2014-049.

### Statistical analysis

Significance was determined by Student’s t-test for comparison amount of *M*. *hominis* in biological samples (a value of *P*<0.01 was considered statistically significant) and Fisher's exact test for comparison *M*. *hominis* positive/negative samples (ϕ*> ϕ_0.01_ was considered statistically significant).

## Results

### Generation and modification of nanobodies to lipid-associated membrane proteins of *M*. *hominis*

**Fig 1:**
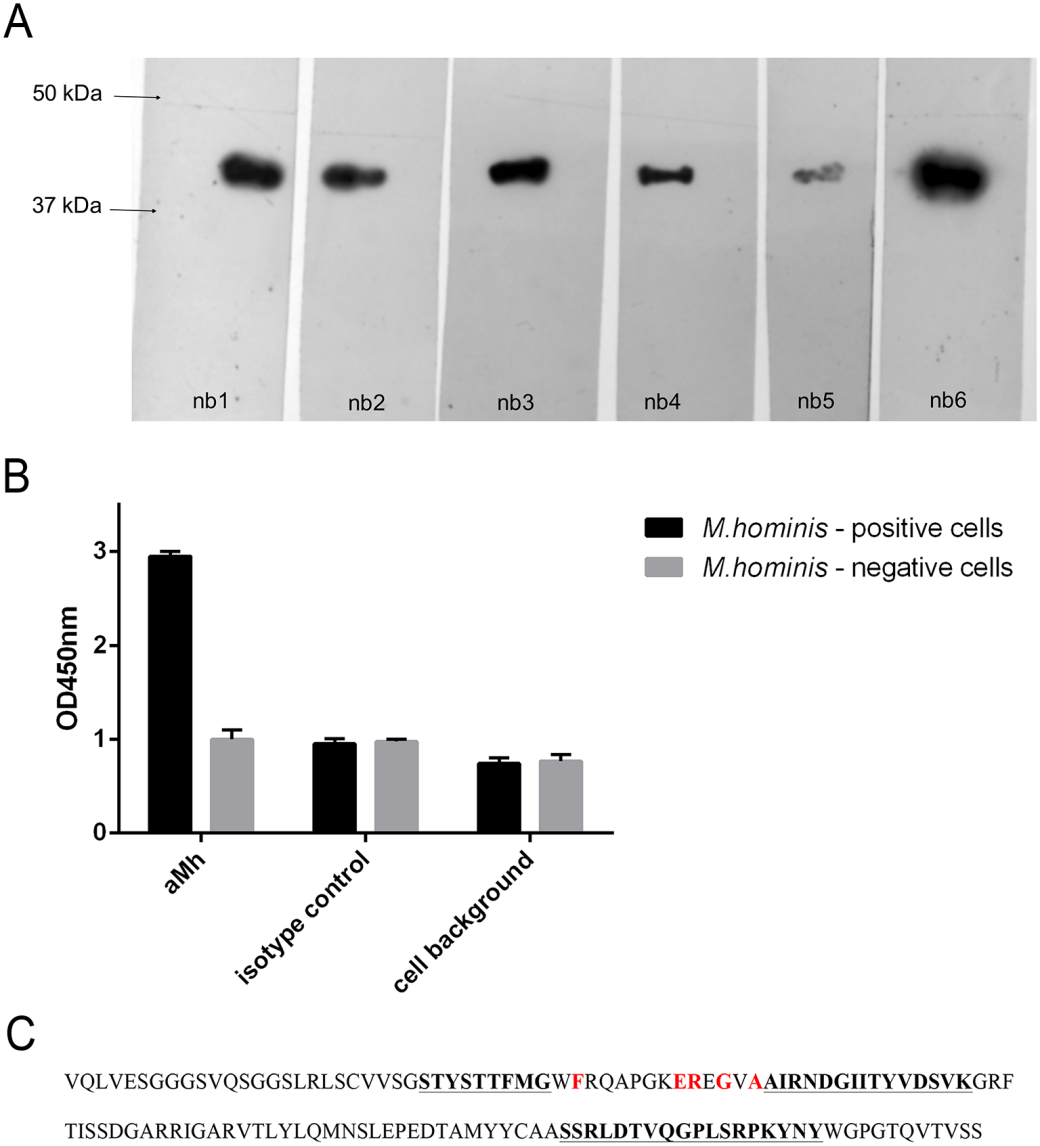
**A**—Western blot analysis of binding selected nanobodies to LAMPs from *M*. *hominis*. LAMPs from *M*. *hominis* H-34 was boiled with Laemmli sample buffer, loaded on 12% Laemmli SDS-PAGE; and Western blot was performed for binding with nanobodies (nb 1–6) at working concentration 1 mkg/ml. **B**—Cell-based ELISA analysis of binding selected nanobody nb 6 (named aMh) to mycoplasma infected cells. *M*. *hominis* positive (experimentally infected) and control *M*. *hominis* negative A549 cells were fixed with paraformaldehyde and ELISA with selected aMh (nb6) was performed. aMh—incubation with aMh, isotype control—incubation with control nanobody, cells background—fixed cells background. **C**—Amino acid sequence of the selected nanobody aMh. Hypervariable regions CDR1, CDR2 and CDR3 are underlined and highlighted in bold. Positions of characteristic amino acids that are specific for single domain antibodies are highlighted in red (these amino acids differ in variable domains of classical type antibodies).

A camel (*C*. *bactrianus*) was immunized with *M*. *hominis* H-34 lipid-associated membrane protein fraction (LAMPs). The end-point ELISA titer of the Camelidae-specific heavy chain-only antibodies against the LAMPs increased more than 100-fold in the immune serum as compared with the preimmune serum (data not shown). Anti-LAMPs nanobodies were then generated, selected and modified as described in the Materials and Methods. After 3 rounds of panning and ELISA-based analysis 6 nanobodies (nb1-6) with different amino acid sequences were identified. The nanobodies were produced and then purified from the bacterial periplasmic fraction. Western blot analysis with LAMPs from *M*. *hominis* shows that all of selected nanobodies bind antigen determinant with Mw ~40–45 kDa (Fig 1). Out of several selected nanobodies, one nanobody variant—the nb6, named aMh (abbreviation from “anti- *Mycoplasma hominis*”)—was considered highly promising according to the highest ELISA binding activity and low background in cell-based ELISA. The aMh nanobody was able to efficiently target mycoplasma-infected cells in a cell-based ELISA with a low background signal (Fig 1). The amino acid sequence of the initially selected nanobody aMh is shown in Fig 1.

The selected nanobody has size about 15 kDa resulting in rapid renal clearance from the organism and weak therapeutic action. Oligomerization of nanobody could significantly increase the *in vivo* half-life [30,37]. Naturally occurring heavy chain antibodies are dimerized through Fc regions of heavy chains. Presence of Fc-region not only determines the dimer formation but also FcR-dependent recycling and antibody effector functions, furthermore, FcR-dependent transcytosis across the mucosal barrier provide immunity to genital pathogens. We used Fc-fusing as a strategy to improve nanobody-based therapeutics for the treatment of genital infection caused by *M*. *hominis*. The selected nanobody-coding sequence aMh was subcloned into the pSh-CMV plasmid vector together with the PLAP leader sequence for secreted protein production, the camel upper hinge (the longest hinge variant) and mouse FcG2a domain sequence. The nanobody-Fc fusion was purified from the culture medium of an A549 cell line transiently transformed with pSh-CMV-PLAP-aMh-FcG2a with the use of protein A resin.

### aMh-FcG2a characterization

**Fig 2:**
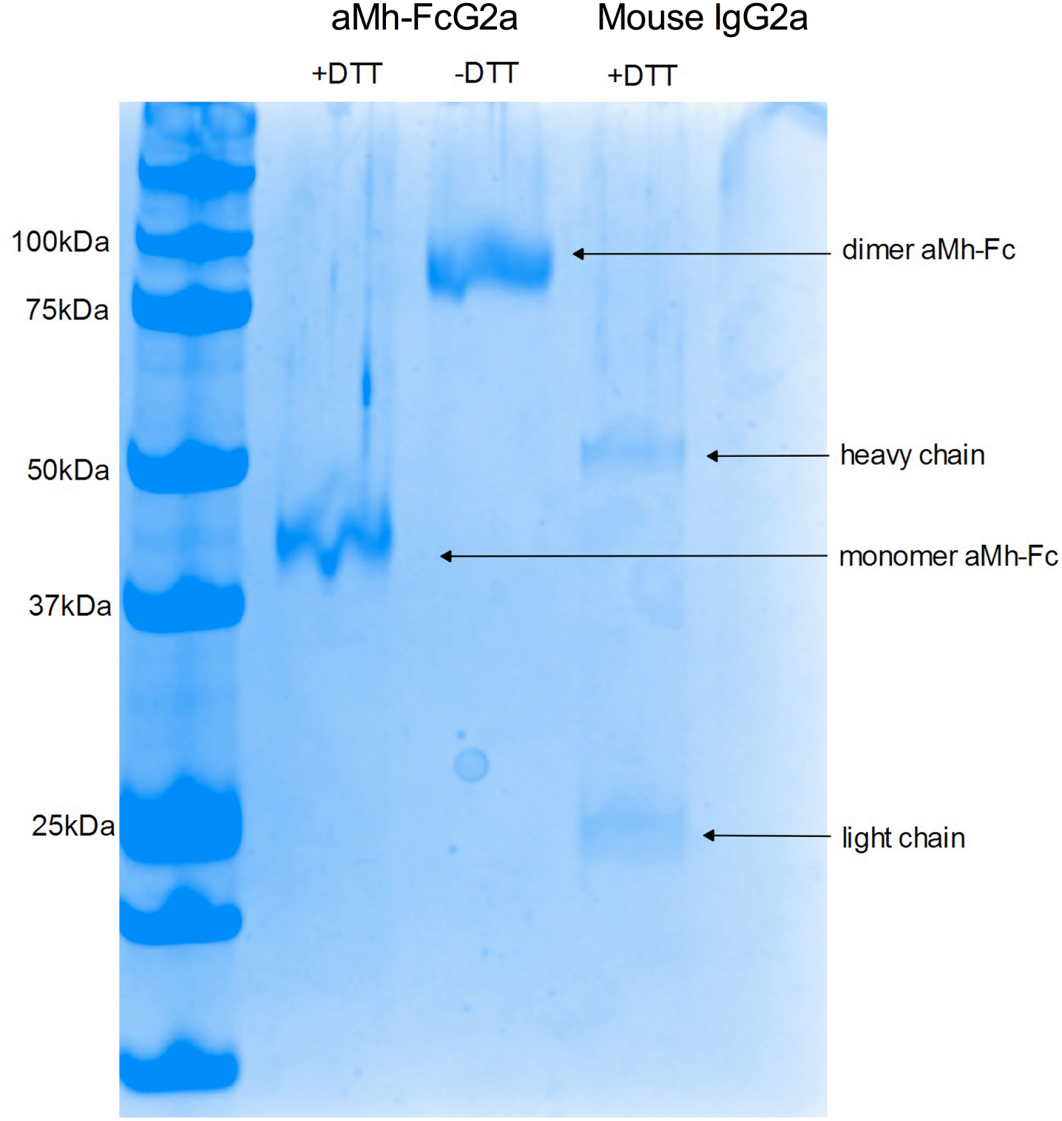
Dimer formations of aMh-FcG2a. Protein samples aMh-FcG2a were prepared before electrophoresis in sample buffer with or without DTT (as reducing agent), canonical mouse IgG2a was prepared with DTT and used as control. Protein bands ~42 and ~84 kDa corresponding monomer and dimer forms of aMh-Fc; protein bands ~54 kDa and 25 kDa corresponding heavy and light chains of canonical mouse IgG2a.

Dimer formation of aMh-FcG2a was verified by electrophoresis in semi-denaturing conditions (Fig 2).

A band of 84 kDa corresponded with dimer formation.

**Fig 3:**
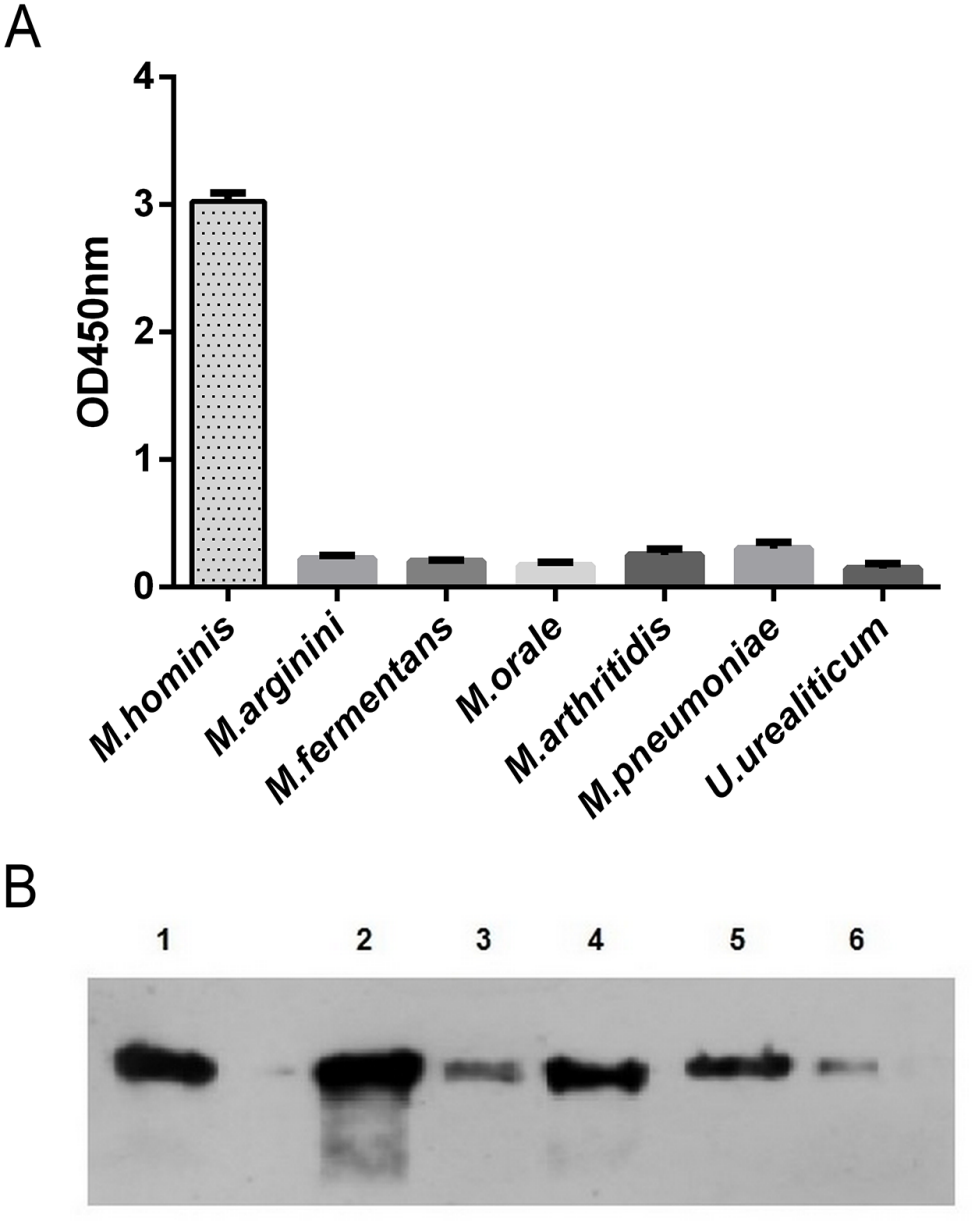
**A**—aMh-FcG2a specificity against different mycoplasma species. LAMPs from different mycoplasma species were coated on the immunoplate at a concentration of 100 ng/well and ELISA was performed with aMh-Fc at working concentration 10nM, BSA was coated at a concentration of 100 ng/well as negative control. **B**—aMh-FcG2a binding with different clinical isolates of *M*. *hominis*. Pellet of *M*. *hominis* cells from 0.1 ml broth culture (titer ~10^6^−10^7^ CCU/ml) was boiled with Laemmli sample buffer, loaded on 12% Laemmli SDS-PAGE. Western blot was performed for binding with aMh-Fc at working concentration 10nM. 1 –reference strain H-34, 2–6 –clinical isolates.

The specificity of aMh-FcG2a was evaluated by ELISA and Western blot with LAMPs from different mycoplasma species (Fig 3A) and different *M*. *hominis* clinical isolates (Fig 3B).

**Fig 4:**
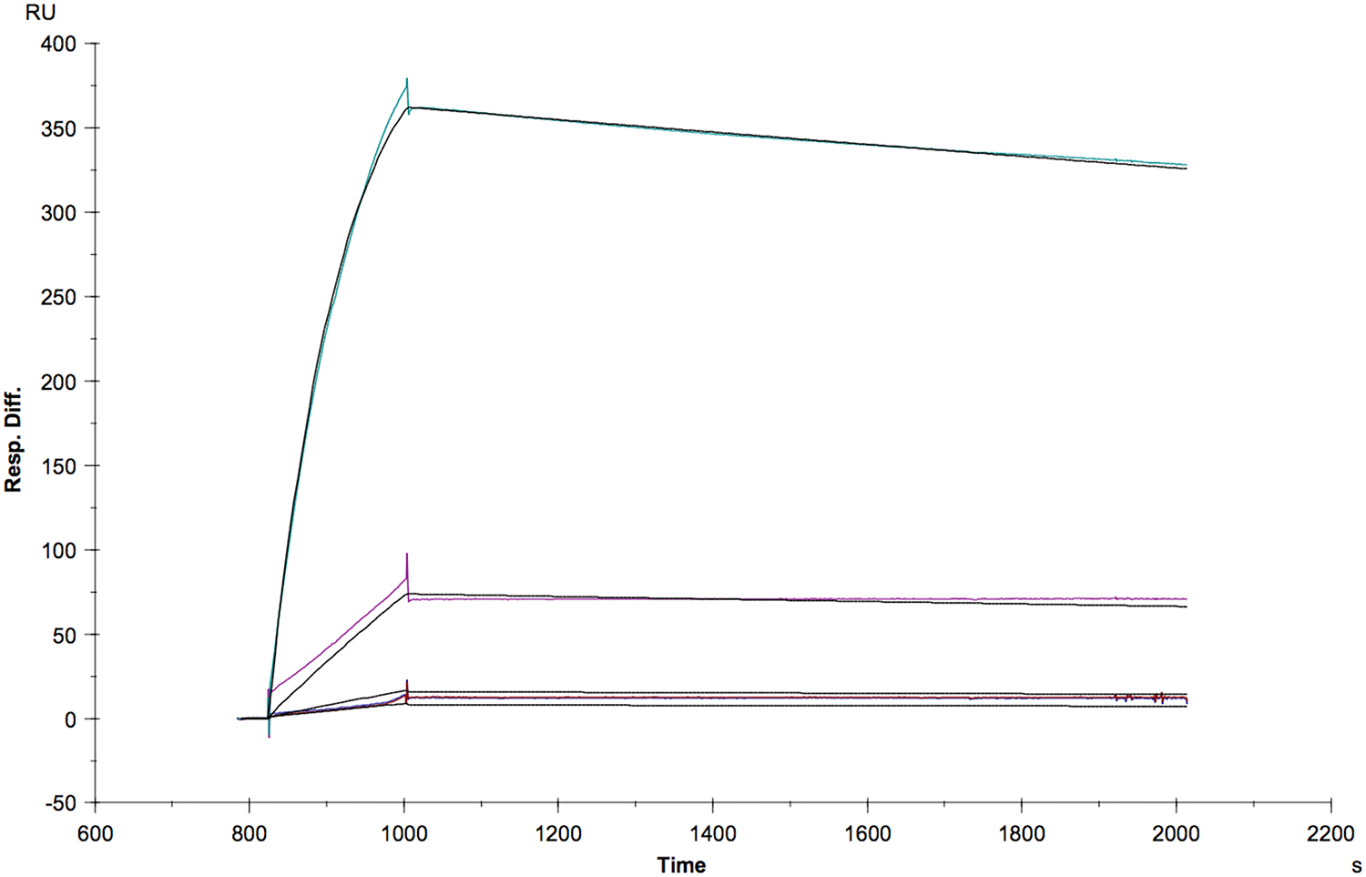
Determination of affinity constants. Binding of antigen to aMh-FcG2a was determined by surface plasmon resonance using Biacore 3000 (GE Healthcare). Antigen concentration series 5.95 nM; 11.9 nM; 59.5 nM and 595 nM (colour) and 1:1 fitting (black) interaction antigen to aMh-Fc. The fitted constant are k_a_ = 1.78^4^ M^-1^s^-1^ and k_d_ = 1.06^−4^ s^-1^ which results *K*_*D*_ = 5.94*10^−9^ M (R_max_ = 428 RU; chi^2^ = 11.8). Evaluation included double reference subtraction.

aMh-FcG2a bound to antigen determinants only from *M*. *hominis* and not from other mycoplasma species, including the closely related *M*. *arginini*. All tested *M*. *hominis* clinical isolates showed the presence of the target antigen. The affinity constants of purified aMh-FcG2a were calculated by measuring SPR imaging units (Fig 4).

Direct antigen immobilization on the surface of CM5 chip leads to loss of the binding activity due to absence of suitable regeneration conditions (10mM glycine-HCl buffers with pH 1.5; 2.0; 2.5; 3.0; 3.5). That`s why we used antibody (anti-mouse IgG) capturing approach for determination of affinity constants. Determined reaction constant is *K*_*D*_ = 5.94 nM.

### Identification of aMh-FcG2a target protein

For antigen identification we precipitated specific targets from LAMPs suspension using magnetic beads coated with aMh-FcG2a. After two rounds of precipitation and purification, as described in methods, protein band was used for identification with matrix-assisted laser desorption/ionization mass spectroscopy (MALDI). MALDI results indicated that aMh-FcG2a bound ABC transporter substrate binding protein (MH3620). MH3620 protein, according to Kegg pathway analysis, is a part of the phosphonate transfer system [38].

### aMh-FcG2a inhibits M. hominis growth *in vitro*

**Fig 5:**
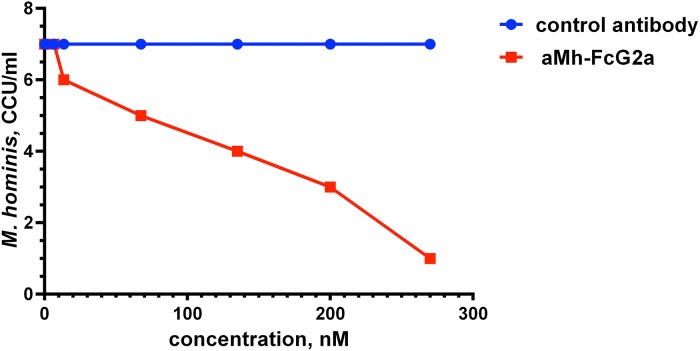
Inhibition of *M*. *hominis* growth with the direct addition of aMh-FcG2a to STE2 medium. aMh-FcG2a was added in different concentrations to *M*. *hominis* inoculated in STE2 medium. *M*. *hominis* titer was determined with CCU counting.

ABC transporter protein complexes are considered to be potential therapeutic targets for anti-mycoplasma drugs. Therefore, we have tested the direct anti-mycoplasma action of aMh-FcG2a. Addition of aMh-FcG2a, but not a control antibody with the same Fc domains, in the culture medium inhibited the growth of *M*. *hominis in vitro* (Fig 5).

### Production of recombinant adenoviral vectors

**Fig 6:**
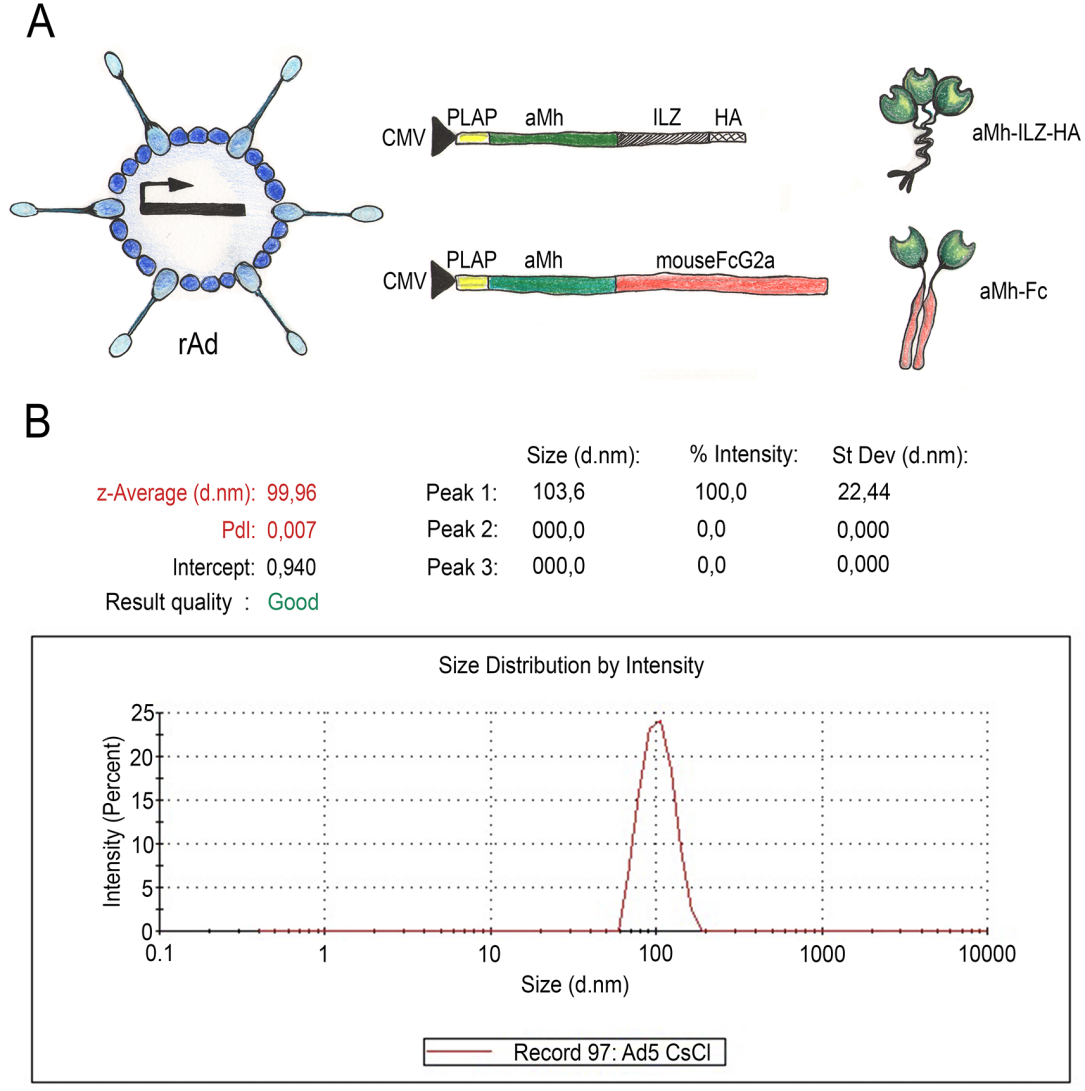
rAd5-CMV-PLAP-aMh-FcG2a and control rAd5-CMV-PLAP-aMh-ILZ-HA constructions and purification. (A) Scheme of expression cassettes for rAd5-CMV constructions; (B) sizing of purified Ad5-CMV-PLAP-aMh-FcG2a corresponding to virions suspensions with a particle size of ~100 nM.

Direct passive immunization for therapy by injecting a protein may fail due to various complications. On the one hand, there is strong mycoplasma compartmentalization in genital tissues. On the other hand, therapy for mycoplasma infection needs long-lasting passive immunity. To overcome this limitation, we obtained adenoviral-based recombinant vectors that can express transgene for up to several weeks. Recombinant adenovirus 5 serotype (rAd5-CMV-PLAP-aMh-FcG2a), expressing aMh-FcG2a, and control rAd5-CMV-PLAP-aMh-ILZ-HA, expressing oligomer forms (mono-, di-, and trimerized) aMh-ILZ-HA, were constructed and produced using the AdEasy vector system. Trimerized form of aMh-ILZ-HA has Mw = 75 kDa, which is closer to aMh-FcG2a dimer (Mw = 84 kDa). Recombinant adenoviruses purified and the size of the particles in suspension verified using a nanosizing device (Fig 6).

**Fig 7:**
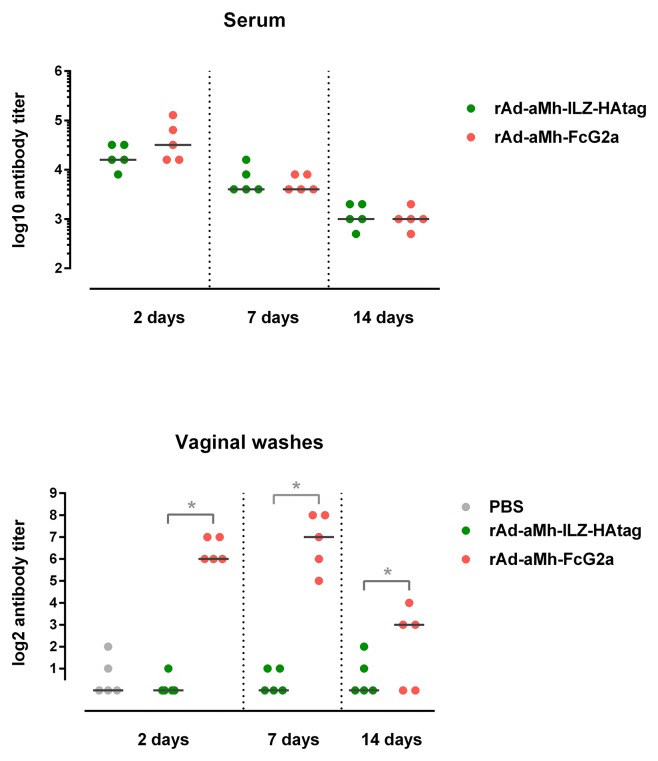
Titer of aMh-FcG2a and aMh-ILZ-HA in serum (A) and in vaginal washes (B) from mouse system inoculated with rAd5-CMV-PLAP-aMh-FcG2a or rAd5-CMV-PLAP-aMh-ILZ-HA. The DBA/2 mice were injected into the ophthalmic venous sinus with 10^7^ PFU of rAd5-CMV-PLAP-aMh-FcG2a or rAd5-CMV-PLAP-aMh-ILZ-HA previously dialyzed against PBS. As a control, we used mice injected with PBS.

Intravenous administration of rAd5 expressing aMh-FcG2a provides significant level of aMh-FcG2a both: in serum and vaginal washes. Presence of aMh-FcG2a in the genital washes and serum of mice was evaluated in 2 (48 h), 7 and 14 days after rAd5-CMV-PLAP-aMh-FcG2a or rAd5-CMV-PLAP-aMh-ILZ-HA inoculation by titration on LAMPs (Fig 7).

Gene therapy approach provides long aMh-FcG2a expression and circulation both in serum and vaginal washes. Although this expression of aMh-ILZ-HA leads to high serum level, but no traces were found in vaginal washes.

### Administration of rAd5 expressing aMh-FcG2a reduce titer of *M*. *hominis in vivo*

**Fig 8:**
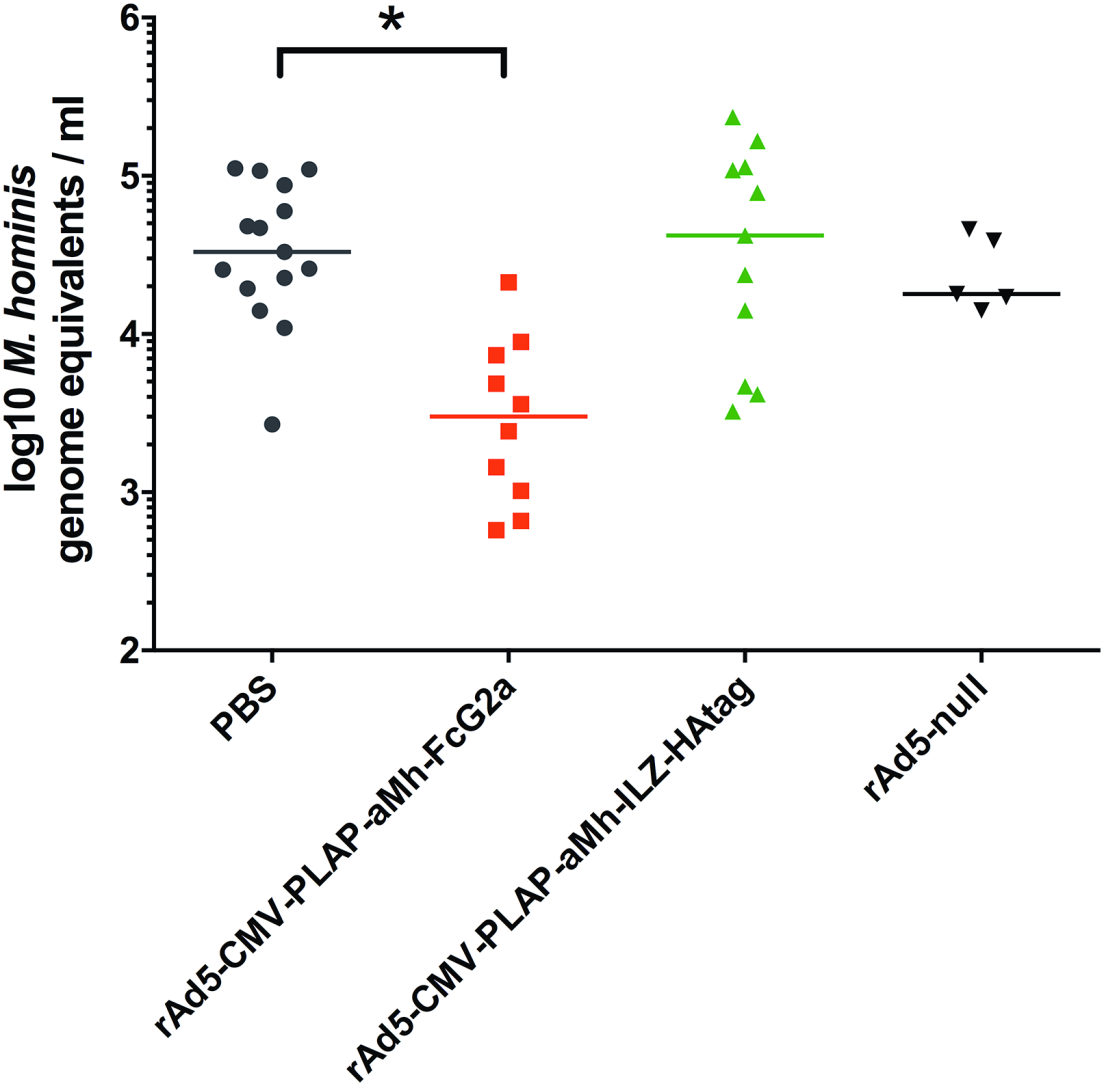
Titers of *M*. *hominis* in vaginal washes with “prophylactic” scheme of rAds inoculation. Vaginal washes were collected 5 days after *M*. *hominis* inoculation (6 days after rAds inoculation). Amount of *M*. *hominis* was evaluated with real-time PCR. The rAd5-CMV-PLAP-aMh-FcG2a group exhibited a significantly lower *M*. *hominis* titer (Student's t-test = 3.5; p<0.01). Ad-null n = 5, PBS n = 15, rAd5-CMV-PLAP-aMh-FcG2a n = 10, rAd5-CMV-PLAP-aMh-ILZ-HA n = 11. In the “prophylactic” scheme of rAds inoculation, the *M*. *hominis* titer was significantly decreased in the rAd5-CMV-PLAP-aMh-FcG2a group. The prophylactic scheme, however, is not practical for treating mycoplasma infection. In the “therapeutic” scheme, the number of animals diagnosed as positive at 7 days after inoculation with rAd5-CMV-PLAP-aMh-FcG2a (12 days after *M*. *hominis* inoculation) was significantly lower than that of the other groups. Nevertheless the mycoplasma titer was not significantly different among the infected animals in any of the groups and not informative due to the large variability in counts and the low number of infected animals in the rAd5-CMV-PLAP-aMh-FcG2a group (n = 2 at 3 days and n = 1 at 7 days).

**Fig 9:**
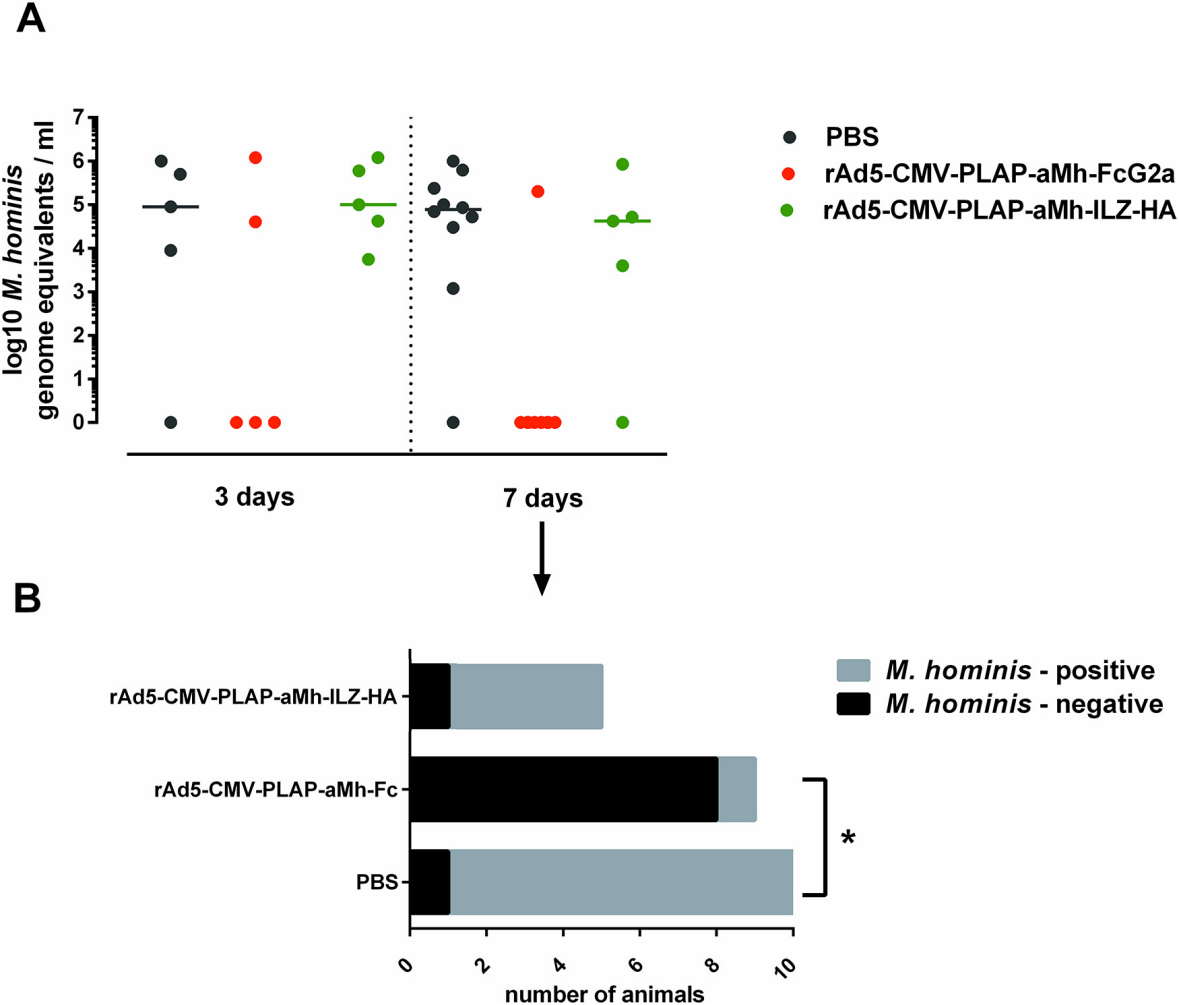
(A) Titers of *M*. *hominis* in vaginal washes with “therapeutic” scheme of rAds inoculation. Vaginal washes were collected 12 days after *M*. *hominis* inoculation (7 days after rAds inoculation). Amount of *M*. *hominis* was evaluated with both CCU and real-time PCR. (B) The same data for 7 days after rAds inoculation through qualitative way (as *M*. *hominis*–positive or *M*. *hominis*–negative sample). The decrease in the number of *M*. *hominis*—positive samples was statistically significant at 7 day after rAd5-CMV-PLAP-aMh-FcG2a inoculation with Fisher's exact test ϕ* = 3.959 (ϕ_0.05_ = 1.64; ϕ_0.01_ = 2.31). The mycoplasma titer was not significantly different among infected animals in the groups due to the large variability in counts and the low number of infected animals in the rAd5-CMV-PLAP-aMh-FcG2a group (n = 2 at 3 days and n = 1 at 7 days).

For an *in vivo* infection model, we used previously established mouse-adapted clinical isolates of *M*. *hominis* 18623 (provided by Professor V. Lazarev from The Scientific Research Institute of Physical-Chemical Medicine, FMBA of Russia) [39]. DBA/2 mice with a previously synchronized estrous cycle, which are highly sensitive to the genital infection strain, were intravaginally infected with 10^6^ CFU *M*. *hominis*. rAd5-CMV-PLAP-aMh-FcG2a, rAd5-CMV-PLAP-aMh-ILZ-HA, rAd5-null or PBS was injected into the ophthalmic venous sinus using one of two schemes: 1 day before (“prophylactic” scheme, Fig 8) or 5 days after infection (“therapeutic” scheme, Fig 9). Vaginal washes collected at the indicated time points were divided into two parts: one for DNA isolation and real-time PCR and the other for microbiologic counting by CCU. The strong correlation between real-time PCR and microbiology-based assays were obtained, but real-time PCR method produced more accurate quantitative data spanning a high-resolution range.

Administration of rAd5-CMV-PLAP-aMh-FcG2a demonstrated both prophylactic and therapeutic effects in a mouse model of genital *M*. *hominis* infection, which is explained by the ability of aMh-FcG2a to penetrate in vaginal fluid due to interaction with FcRn.

## Discussion

Antibody treatment of infectious diseases is one of the most promising trends in medicine and life science. Unlike some virus pathogens and bacterial toxins, treatment of bacterial infections requires long-lasting passive immunity, and anti-bacterial activity must include both direct pathogen opsonization and the involvement of complex mechanisms such as cell-based immune reactions [18,40].

Nanobodies are highly promising molecules based on the antigen-binding variable fragment (VHH) of the heavy chain-only unconventional antibody found in *Camelidae*. Heavy chain antibodies evolved to compensate for the absence of a light chain partner and have a unique structure of complementary determining regions (CDRs), especially CDR3. CDR3 often comprises a long loop and could have the unique ability to bind small protein cavities or enzyme clefts. This feature of nanobodies leads to the great potential for nanobody-mediated inactivation of specific targets [20,24,41]. Isolated nanobody aMh specifically binds to ABC-transporter substrate-binding protein. The aMh show direct mycoplasmocidal action and give occasion to speculate about potential function and mode of aMh action. Antagonisms of nutrient acquisition through ABC transporters are reported for *Staphylococcus aureus* (Fab fragment [42] and recombinant antibody Aurograg^®^ (Novartis)) and *Streptococcus mutans* [43].

**Fig 10:**
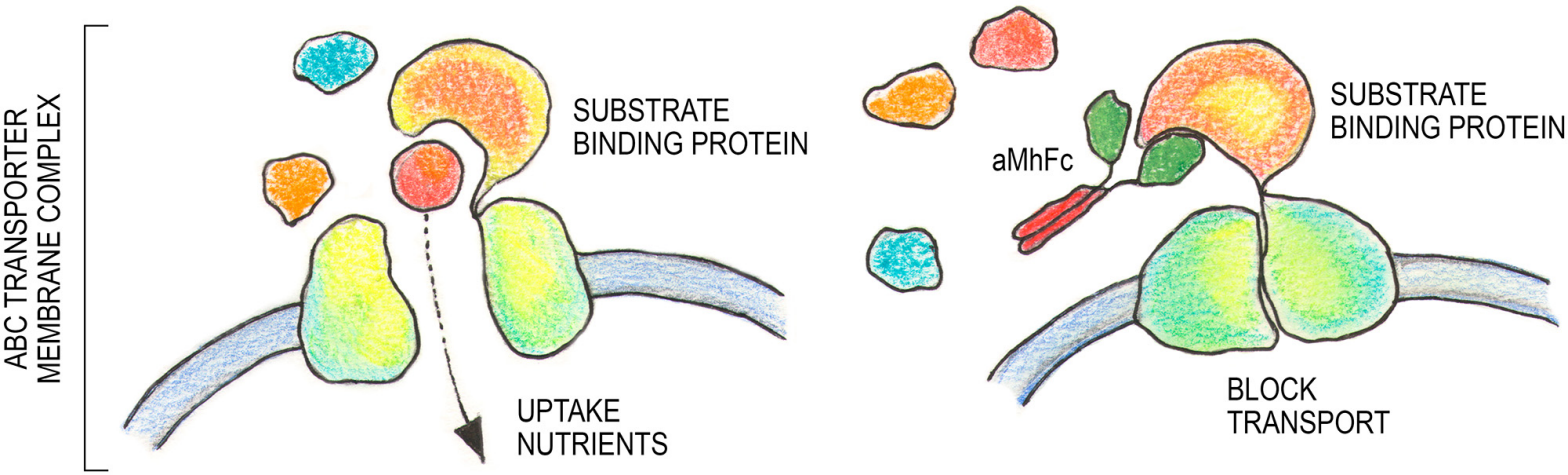
Hypothetical mechanism of blocking nutrient acquisition through ABC transporter with aMh-FcG2a.

Thus, following the reasoning set forth by the influence of aMh on transport function, we hypothesize that aMh binding to substrate-binding protein blocks nutrients uptake and inhibit mycoplasma growth (Fig 10).

The practical application of nanobody fragments has been limited by problems related to rapid blood clearance and poor retention time at the target, thus requiring frequent delivery of these molecules and the absence of the Fc domain for complex immune reactions. The single-chain nature of nanobodies offers great potential for its genetic delivery and construction of fusion proteins. Genetically delivered nanobodies are effective for therapeutic applications against flu, botulism and infection with Shiga toxin-producing *E*. *coli* [29,30,44]. Adenoviral vectors are widely used as gene therapy vectors due to higher transduction efficiency, higher level expression of transgene and safety. Replication-deficient adenoviral vectors deliver their genomes into the nucleus of target cells where they form episomes. After intravenous injection the vast majority adenoviral vectors are localized in liver and the minority in spleen and kidneys [45]. Experiments *in vivo* showed that adenoviral vectors remained for a temporal period about 10 weeks with the maximum transgene expression at 48–96 hours [46]. *In vivo* production mediated by adenovirus-based gene therapy vectors results in effective and persistent levels of nanobodies and promotes long-lasting passive immunity [29,30,44].

In most cases local or systemic delivery of nanobody-expressing vectors results in significant protection only if the pathogens or toxins have the same distribution in the organism. Thus, the treatment of genital infections requires intravaginal drug administration, which is probable due to the barrier function of cervical mucus. At the same time, FcRn-dependent endogenous antibodies transcytosis across the mucosal barrier provide local immunity against various pathogens [47–50]. Surprisingly, only a few studies examined whether Fc-fusion could be used for transferring nanobodies via naturally existing mechanisms [51,52]. Moreover, Fc-mediated reactions are significant for development of protective immunity to pathogens: destruction by phagocytes following opsonization of free particles or infected cells by antibodies or by natural killer cells via FcR-dependent antibody-dependent cell-mediated cytotoxicity [32,51–54]. We used Fc-fusing as a strategy to improve nanobody-based therapeutics for the treatment of genital infection caused by *M*. *hominis*. Additionally, species-specific Fc replacement can potentially be used as treatment for human infections.

We demonstrated that the fusion of aMh with the Fc fragment of mouse IgG2a provided an efficient transfer chimeric aMh-Fc from the systemic circulation to the genital mucus. Our results revealed that the aMh, a nanobody selected against LAMPs bound to an ABC transporter substrate binding protein (MH3620), inhibited the growth of *M*. *hominis in vitro*. Adenoviral-based vector for passive immunization expressing aMh-Fc chimeric antibodies combine such advantages as the gene-therapy approach, specific anti-mycoplasma nanobody action and Fc-mediated transfer to the genital mucus.

## Conclusion

In summary, we have developed a novel therapeutic approach for *M*. *hominis* infection using adenoviral vector-based genetic passive immunization expressing the nanobody-Fc chimeric drug. This study combines the advantages of gene-therapy, specific nanobody properties and Fc-mediated reactions. A comprehensive approach may overcome limitations and have great potential for therapy against bacterial infection using nanobodies.
